# Neuromicrobiology, an emerging neurometabolic facet of the gut microbiome?

**DOI:** 10.3389/fmicb.2023.1098412

**Published:** 2023-01-17

**Authors:** Saba Miri, JuDong Yeo, Sarah Abubaker, Riadh Hammami

**Affiliations:** ^1^School of Nutrition Sciences, Faculty of Health Sciences, University of Ottawa, Ottawa, ON, Canada; ^2^Department of Biochemistry, Microbiology and Immunology, Faculty of Medicine, University of Ottawa, Ottawa, ON, Canada

**Keywords:** gut-brain axis, gut microbiome, microbial neurometabolites, neurotransmitter, GABA, SCFAs, dopamine, serotonin

## Abstract

The concept of the gut microbiome is emerging as a metabolic interactome influenced by diet, xenobiotics, genetics, and other environmental factors that affect the host’s absorption of nutrients, metabolism, and immune system. Beyond nutrient digestion and production, the gut microbiome also functions as personalized polypharmacy, where bioactive metabolites that our microbes excrete or conjugate may reach systemic circulation and impact all organs, including the brain. Appreciable evidence shows that gut microbiota produce diverse neuroactive metabolites, particularly neurotransmitters (and their precursors), stimulating the local nervous system (i.e., enteric and vagus nerves) and affecting brain function and cognition. Several studies have demonstrated correlations between the gut microbiome and the central nervous system sparking an exciting new research field, neuromicrobiology. Microbiome-targeted interventions are seen as promising adjunctive treatments (pre-, pro-, post-, and synbiotics), but the mechanisms underlying host-microbiome interactions have yet to be established, thus preventing informed evidence-based therapeutic applications. In this paper, we review the current state of knowledge for each of the major classes of microbial neuroactive metabolites, emphasizing their biological effects on the microbiome, gut environment, and brain. Also, we discuss the biosynthesis, absorption, and transport of gut microbiota-derived neuroactive metabolites to the brain and their implication in mental disorders.

## 1. Introduction

Over the past few decades, increasing attention has been paid to the gastrointestinal microbiome as one of the key elements contributing to the regulation of host physiology (de Vos et al., 2022). The microbiome has recently been redefined to pertain not only to the community of microorganisms but also their theatre of activity, including microbial structures, metabolites, and mobile genetic elements, whereas the microbiota is an assemblage of microbial communities associated with a habitat (Berg et al., 2020). The metabolic activities of gut symbionts go beyond simply assisting in digestion and nutrient production, or modulating and protecting the intestinal barrier, and have important implications for one health (Berg et al., 2020). Over the past decade, gut neuromicrobiology has emerged as an exciting area of research that encompasses understanding the link between the gut microbiome, its neurometabolic interactome, and its association with brain health and diseases (de la Fuente-Nunez et al., 2018). Indeed, appreciable evidence highlight that alterations in the diversity and the metabolic activity of the gut microbiome, also known as “dysbiosis,” are linked to multiple psychiatric and neurological disorders (de la Fuente-Nunez et al., 2018).

**Figure 1:**
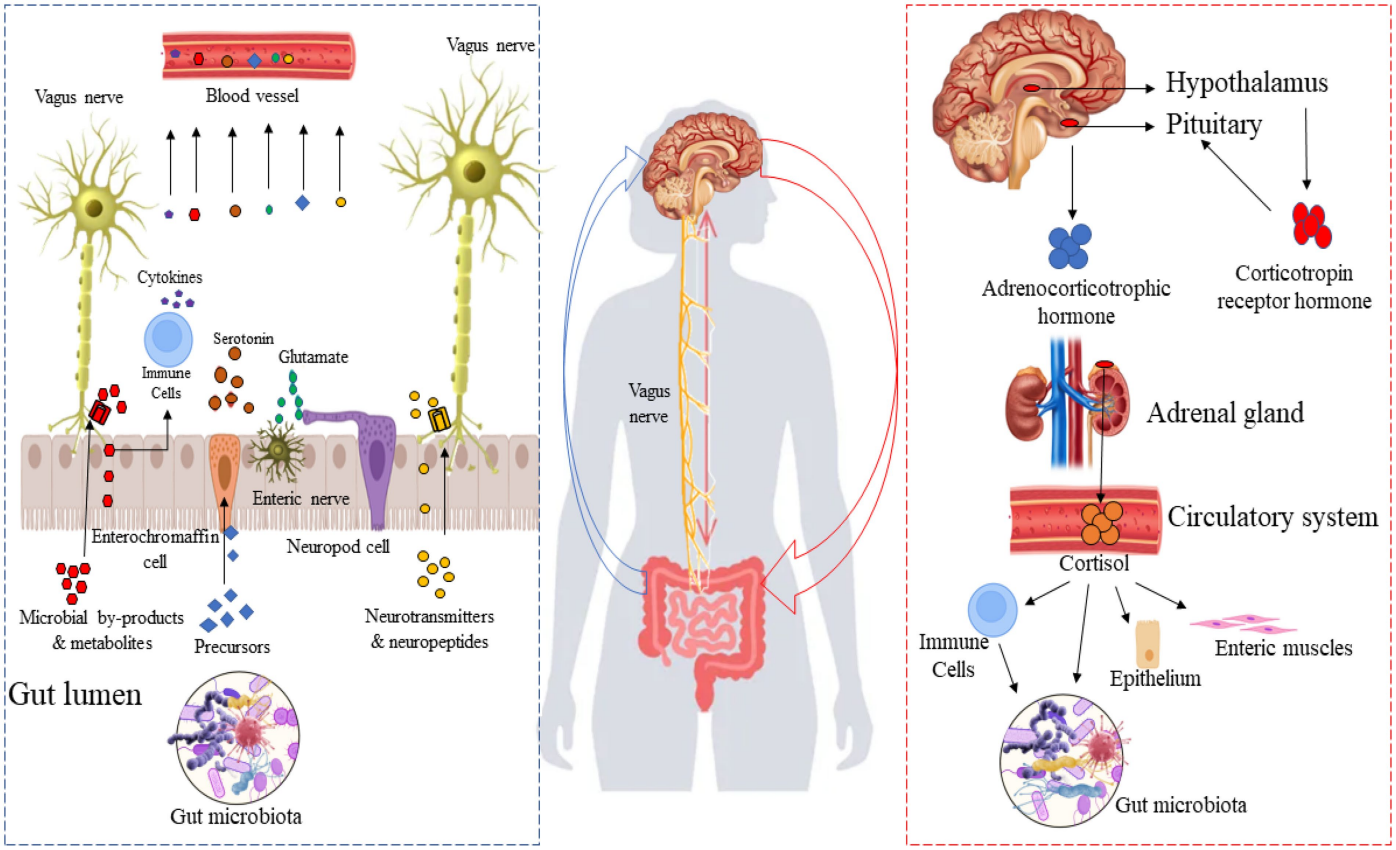
Top-down and bottom-up pathways between the gut microbiota and the brain. Right side: Gut microbiota-derived neurotransmitters and their precursor in the gut microbiome-brain axis; left side: the hypothalamus-pituitary–adrenal axis.

The gut-brain axis is a bi-directional communication system linking the gut microbiome to the brain and plays a crucial role in neuronal development, cognitive regulation, mental state, emotional regulation, behavior, and brain function (Cryan et al., 2020; Agirman and Hsiao, 2021). Gut-brain axis activity can be modulated by broadly two approaches: “top-down” and “bottom-up” (Figure 1). A combination of endocrine (cortisol), immune (cytokines), and neural (vagus and enteric nervous systems) pathways are involved in these two approaches. In the top-down approach, the brain recruits these mechanisms in order to influence the composition of the microbiota in the gut. It is known that the hypothalamus-pituitary–adrenal axis regulates cortisol secretion under stress conditions, and cortisol directly affects immune cells (including the secretion of cytokines) both locally in the gut and systemically. Also, cortisol affects gut permeability and barrier function, as well as the composition of the gut microbiota (Cryan and Dinan, 2012). In the bottom-up approach, the gut microbiota signals the brain through immune regulation (production of cytokines) and the production of microbial neuroactive metabolites and neurotransmitters. Through this approach, for instance, the level of systemic tryptophan and the stimulation of the vagus and enteric nerves play a significant role in the communication between the gut microbiome and the brain. Appreciable evidence suggests that the gut microbiota produce a broad spectrum of neuroactive metabolites (Valles-Colomer et al., 2019; Lai et al., 2021), particularly neurotransmitters and their precursors, highlighting a potential involvement in neuroendocrinology-based mechanisms, illustrated by the bottom-up pathway in Figure 1. For example, spore-forming bacteria secrete their metabolites, stimulating serotonin biosynthesis in enterochromaffin cells (Yano et al., 2015). Moreover, some neurotransmitters and their precursors produced by the gut microbiota and enteroendocrine cells are transferred to the bloodstream and could reach the brain. Figure 1 shows the importance of the microbiome and produced neuroactive metabolites in the gut-brain axis, especially in the “bottom-up” pathway.

In recent years, an increasing number of studies have reported on the biosynthesis of gut microbiome-derived neurotransmitters [i.e., γ-aminobutyric acid (GABA), serotonin, dopamine, norepinephrine, etc.] and other neuroactive metabolites that could impact brain functions and condition (Cox and Weiner, 2018; Cryan et al., 2020). For instance, some research groups found that gut dysbiosis and the following interference in releasing monoamine cause severe major depressive disorder (MDD) in an animal model, proving a deep relationship between the gut microbiome and mental disorders (Heijtz et al., 2011; Neufeld et al., 2011; Clarke et al., 2013). Therefore, microbially-produced neuroactive metabolites could be an integral part of the gut microbiome–host crosstalk mechanisms, thus, eliciting various health-promoting effects. Despite recent research progress, multiple questions surrounding gut neuromicrobiology remain unsolved. Why and how do some specific gut microbes harbor the genes responsible for producing neuroactive molecules but not others? Is it an intra-kingdom or inter-kingdom quorum sensing signaling mechanism or both? What are the possible routes of delivery of these neuroactive metabolites to the gut environment and brain? In this review, we discuss the diversity, biosynthesis, transport, and interplay of microbiome-produced neuroactive metabolites with the gut-brain axis.

## 2. Microbiota-produced neurotransmitters and related metabolites

### 2.1. Diversity within gut neurotransmitter-producing bacteria

**Figure 2:**
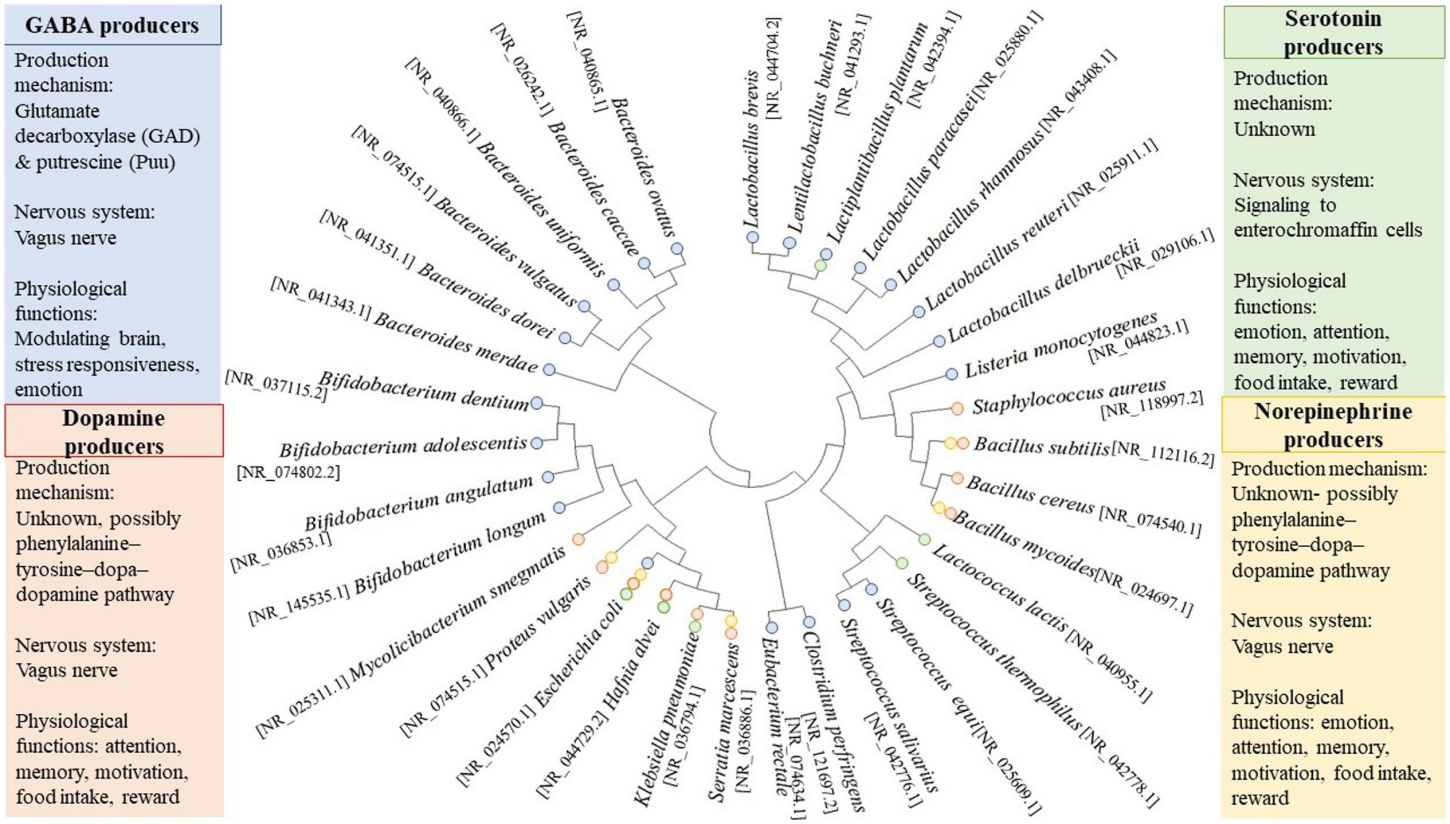
Phylogenetic diversity of neurotransmitter-producing bacteria. Sequences are based on published whole-genome or partial sequences from the NCBI Reference Sequence (NCBI RefSeq Targeted Loci Project, Direct Submission, National Center for Biotechnology, Information, NIH, Bethesda, MD 20894, USA). GeneBank accession numbers for the 16S rRNA sequence are shown in the bracket. The phylogenetic tree was constructed using MEGA 11 software (version 11.0.10). Briefly, the evolutionary history was inferred using the Neighbor-Joining method (Saitou and Nei, 1987). The bootstrap consensus tree inferred from 1,000 replicates represents the evolutionary history of the taxa analyzed (Felsenstein, 1992). Branches corresponding to partitions reproduced in less than 50% of bootstrap replicates are collapsed. The evolutionary distances were computed using the Tamura 3-parameter method (Tamura, 1992) and are in the units of the number of base substitutions per site. This analysis involved 34 nucleotide sequences. All ambiguous positions were removed for each sequence pair (pairwise deletion option). There were a total of 1,606 positions in the final dataset. Evolutionary analyses were conducted in MEGA11 (Tamura et al., 2021).

A consideration of some of the more well-studied neuroactive gut microorganisms demonstrates their considerable phylogenetic and neuroactive diversity (Figure 2). As detailed below, multiple neurotransmitters secreted by the gut microbiome have been reported; as such, gut neuromicrobiology has been proposed as a separate field of study in recent years. As shown in Figure 2, some bacterial strains can produce more than one main neurotransmitter. It is often difficult to correlate neurotransmitter production with phylogeny (Figure 2) due to the possible adaptation of bacteria through horizontal gene transfer. Indeed, the gut environment is one of the most favorable ecological niches for lateral gene transfer, which is characterized by stable temperatures, continuous food supply, stable physicochemical conditions, a high concentration of bacterial cells and phages, and ample opportunities for conjugation of these cells and phages on food particles and host tissues (Lerner et al., 2017). In response to selective pressures in the gut, bacteria may undergo genetic restructuring, but the transfer of neuroactive genes has not yet been documented so far.

### 2.2. Synthesis of neurotransmitters by gut microbiota

#### 2.2.1. γ-Aminobutyric acid

GABA, a nonprotein amino acid generated by the decarboxylation of glutamic acid, is a naturally occurring amino acid, and it functions as a neurotransmitter at the inhibitory synapses of the vertebrate and invertebrate nervous system. GABA plays a crucial role in controlling neuronal excitability in the nervous system and has shown many other physiological functions. It is important to mention that a wide range of GABA-binding proteins are present in gut-associated bacteria and are thought to be critical in bacterial and inter-domain communication (Valles-Colomer et al., 2019). The low level of GABA in the brain causes severe psychiatric and neurological disorders, including depression, anxiety, insomnia, and epilepsy (Luscher et al., 2011; Gabbay et al., 2017; Erjavec et al., 2021). Some evidence revealed that the gut microbiome affects the level of GABA and subsequently influences mental health. For instance, Bravo et al. (2011) reported that *L. rhamnosus* elevated the abundance of GABA_B1b_ mRNA (GABA_B_ produces slow and prolonged inhibitory signals) while decreasing the level of GABAA_α2_ mRNA (GABA_A_ mediates fast inhibitory signals) in the cortex of mice, leading to the inhibition of anxiety and depression-like behaviors (Bravo et al., 2011; Terunuma, 2018). In mammalians, approximately 25–50% of neurons contain GABA as a primary inhibitory neurotransmitter in their central nervous system (CNS; Peters et al., 2019). In this section, we focused on the biosynthesis of GABA in potential gut microbes. The biosynthesis of GABA has been reported in various microorganisms (Figure 2). Microbial species can produce GABA either using the glutamate decarboxylase (GAD) or putrescine (Puu) pathways (Diez-Gutiérrez et al., 2020). Most bacteria use the GAD pathway, while the Puu pathway is considered a minor route for synthesizing GABA (Diez-Gutiérrez et al., 2020). Mainly, *Lactobacillus* spp., *Bifidobacterium* spp., *Escherichia coli*, *Listeria monocytogenes*, and *Aspergillus oryzae* produce GABA through the GAD pathway (Huang et al., 2014; Das and Goyal, 2015; Sano et al., 2016), while the Puu pathway is described only for *Escherichia coli* (Cha et al., 2014) and *Aspergillus oryzae* (Akasaka et al., 2018). The GAD pathway is initiated by Glu/GABA antiporters encoded by a *gadC* gene (Gao et al., 2019). As a result of the action of this antiporter, glutamate or monosodium glutamate is pumped into the microorganism (Choi et al., 2013). *gadB* gene encodes the GAD enzyme, which catalyzes the transformation of Glu to GABA. This enzyme consists of six repetitive subunits containing a conserved lysine residue that binds to pyridoxal-5-phosphate (Yu et al., 2019). However, Lyu et al. (2019) reported that the *gadA* gene plays the same role as *gadB* in GAD expression, while the deletion of *gadB* has more effect on reducing GABA production (Lyu et al., 2019).

The putrescine pathway begins with the transport of Puu into the cell *via* an antiporter encoded by the *puuP* gene (or *ycjJ*). Then, Puu undergoes two paths; (1) direct conversion to γ-aminobutyraldehyde catalyzed by a Puu-amino-transferase encoded by *patA* gene (*ygjG*) and subsequent oxidation to GABA by a γ-aminobutyraldehyde-dehydrogenase encoded by *patD* gene (*ydcW* gene). (2) Transformation to γ-glutamyl-Puu catalyzed by γ-glutamate-putrescine-synthetase encoded by a *PuuA* gene and then two oxidation reactions for the production of γ-Glu-GABA by γ-Glutamyl-oxidase and a γ-glutamyl-γ-butyraldehyde dehydrogenase encoded by *puuB* (*ycjA*) and a *puuC* genes, respectively. Then, γ-Glu-GABA hydrolase (encoded by *puuD* gene) degrades γ-Glu-GABA into GABA (Wu et al., 2017). It is noteworthy that GABA can degrade by following the Puu pathway and entering the tricarboxylic acid cycle (TCA). In this path, GABA converts to succinic semialdehyde catalyzed by GABA-aminotransferase (encoded by *gabT* gene) and subsequently converted into succinate yield by a succinic semialdehyde dehydrogenase encoded by a *gabB* gene (Yu et al., 2019). Then the succinate is introduced into the TCA cycle (Kurihara et al., 2010).

GABA shunts and polyamine pathways are metabolic pathways that enable microorganisms to produce and maintain optimal levels of GABA (Cui et al., 2020). Some gut commensal microbes produce GABA, such as *Bacteroides*, *Bifidobacterium,* and *Lactobacillus* genera, as listed in Figure 2. Strandwitz et al. (2019) reported several GABA-producing bacteria, including *Bacteroides caccae*, *Bacteroides vulgatus*, *Bacteroides ovatus*, *Bacteroides dorei*, *Bacteroides uniformis*, *Parabacteroides merdae*, *Bifidobacterium adolescentis*, and *Eubacterium rectale* in which they showed a discrepancy in GABA-producing capacity depending on pH of the liquid medium used for growing those bacteria, with *B. caccae*, *B. vulgatus*, and *B. ovatus* being the most GABA producers (Strandwitz et al., 2019). Recently, Sultan et al. (2022) reported a high GABA production (3–6 mM) for *B. finegoldii, B. caccae, and B. faecis*, three human gut isolates having a distinctive signature operon compared to low GABA-producing isolates. Previously, Barrett et al. (2012) reported on the GABA-producing capacity of *Lactobacillus* and *Bifidobacterium* from the human gut. Out of 91 tested bacteria, the authors found one *Lactobacillus* strain and four strains of *Bifidobacterium* capable of producing GABA, with *Levilactobacillus brevis* DPC6108 being the most potential producer strain (Barrett et al., 2012). Likewise, Pokusaeva et al. (2017) reported that commensal *Bifidobacterium dentium* generates GABA through the enzymatic decarboxylation of glutamate by glutamate decarboxylase beta (gadB) in the rat fecal retention model (Pokusaeva et al., 2017). Besides, chronic treatment of mice with *Lacticaseibacillus rhamnosus* attenuates depression and anxiety-like behavior by producing GABA and regulating GABA receptors such as GABA_Aα2_ and GABA_B1b_ in the brain (Bravo et al., 2011). Aside from the above microorganisms, several lactobacilli, *Monascus purpureus*, and *Streptococcus salivarius* subsp. *thermophilus* have also been reported as efficient GABA-producing microbes in the gut environment (Cui et al., 2020). A recent study showed that *Lentilactobacillus curieae* produces GABA through two distinct pathways: (1) Transamination of succinic semialdehyde by GABA transaminase; and (2) decarboxylation of L-glutamate by 5-Oxopent-3-ene-1,2,5-tricarboxylate decarboxylase (HpaG; Xie et al., 2022).

#### 2.2.2. Dopamine

Dopamine, 3,4-dihydroxyphenethylamine, is a primary catecholaminergic neurotransmitter that plays a significant role in brain physiological functions (i.e., emotion, attention, memory, motivation, food intake, and reward; Kleinridders and Pothos, 2019). Dopamine dysregulation was strongly associated with psychiatric and neurological disorders, such as anxiety, depression, autism, Parkinson, and Alzheimer’s (Moraga-Amaro et al., 2014; Bäuerl et al., 2018; Eltokhi et al., 2020). Although the brain is the main site of dopamine synthesis, enteric neurons and intestinal epithelial cells produce approximately 50% of total dopamine in the gastrointestinal tract (Eisenhofer et al., 1997). The mechanism of dopamine synthesis is well-known through the phenylalanine–tyrosine–dopa–dopamine pathway. In this pathway, L-phenylalanine is converted to L-tyrosine by phenylalanine hydroxylase, which mainly occurs in the liver and kidney (Møller et al., 2000). L-tyrosine (from the diet or the liver and kidney) can cross the blood–brain barrier (BBB) and enter the brain. In the brain, it converts to (s)-3,4-dihydroxyphenylalanine (L-dopa) by tyrosine hydroxylase, then the transformation of L-dopa is completed to dopamine by dopa decarboxylase (Seeman, 2010). Tyrosine hydroxylase is considered one of the most important enzymes due to its role as the rate-limiting enzyme in the biosynthesis of catecholamines. It is a monooxygenase that contains iron and requires tetrahydrobiopterin (BH_4_) as a cofactor (Nagatsu et al., 2019). There is growing evidence pointing out that the intestinal microbiome contains bacteria that produce BH_4_ and that phenylalanine–tyrosine–dopa–dopamine metabolic pathways also exist in microorganisms. Therefore, bacteria may contain homologs of the enzyme genes that mammals use to produce dopamine (Iyer and Ananthanarayan, 2008; Belik et al., 2017). As shown in Figure 2, several bacteria have been reported to produce dopamine in the gut, including bacilli, *E. coli*, *Proteus vulgaris*, *Serratia marcescens*, *Staphylococcus aureus*, *Hafnia alvei*, *Klebsiella pneumoniae* (Tsavkelova et al., 2000; Cryan and Dinan, 2012). However, the detailed mechanism of dopamine biosynthesis by the gut microbiome has not yet been fully elucidated.

#### 2.2.3. Serotonin

Serotonin, a monoamine neurotransmitter, is involved in various brain functions such as modulating mood, reward, cognition, memory, learning, and many physiological processes, including vasoconstriction and vomiting (Berger et al., 2009). The altered expression, production, and function of serotonin in the brain result in the pathogenesis of mental illnesses, such as anxiety and depressive disorders (Helton and Lohoff, 2015). Several local effects are also conferred by gut-produced serotonin (5-hydroxytryptamine), including stimulating gut motility. The primary serotonin synthesis pathway occurs *via* enteric enterochromaffin cells, in which tryptophan hydroxylase 1 (Tph1) takes part in the reaction as the rate-limiting enzyme for serotonin synthesizing (Kwon et al., 2019). Indeed, most serotonin is present around enterochromaffin cells in the gastrointestinal tract and enteric nerves after their biosynthesis from tryptophan (Spiller, 2008; Gershon, 2013; Mawe and Hoffman, 2013). The production capacity of serotonin by the enterochromaffin cells is beholden to the available level of tryptophan needed for the synthesis; thus, maintaining the abundant amount of tryptophan in the gastrointestinal tract is crucial to synthesize an adequate level of serotonin. So far, many research groups have explored serotonin-producing bacteria in the gut, including *E. coli* K-12, *Lactiplantibacillus plantarum* FI8595, *Lactococcus lactis* subsp. *cremoris* MG 1363, *Streptococcus thermophilus* NCFB2392, *Candida* spp., *Streptococcus* spp., *Escherichia* spp., and *Enterococcus* spp. (Shishov et al., 2009; Cryan and Dinan, 2012). As opposed to eukaryotes, little is known about the serotonin synthesis pathway in bacteria. Several bacteria have been identified to encode for eukaryote-like aromatic amino acid hydroxylase and aromatic amino acid decarboxylase, although the serotonin production pathway has not yet been investigated in most of these bacteria (Gonçalves et al., 2022).

Gut microbiota also indirectly take part in the production of serotonin: for instance, enterochromaffin cells produce serotonin once they receive signals through gut microbiome-produced metabolites that upregulate expression of the *tph1* gene (Legan et al., 2022). Indeed, germ-free mice (GF) have substantially reduced colonic Tph1 mRNA expression, serum serotonin levels, and increased serotonin-selective reuptake transporter mRNA expression compared to control mice (Sjögren et al., 2012). In another study, gut microbiome was shown to play a role in the production of serotonin by comparing three mice groups: GF mice, GF mice colonized with human gut bacteria, and normally raised mice with mouse microbiomes. The colonized mice with human gut bacteria and normally raised mice expressed higher levels of colonic Tph1 mRNA and protein along with an increase in colonic serotonin level compared to GF mice. There was no difference in enterochromaffin cell density between the three groups, so the gut microbiome could directly regulate serotonin levels in the gastrointestinal tract (Reigstad et al., 2015). Likewise, the gut microbiome release short-chain fatty acids and bile acids, inducing serotonin production in the enterochromaffin cells (Reigstad et al., 2015; Legan et al., 2022). Although Legan et al. (2022) provided some evidence of the direct and indirect effects of the gut microbiome on host serotonin systems, they also mentioned that no serotonin-producing human commensal has not yet been reported (Legan et al., 2022).

#### 2.2.4. Norepinephrine

Norepinephrine is a catecholamine that plays roles in learning, attention, cognition, and memory, in addition to its function in alertness, arousal, and sensory detection (Borodovitsyna et al., 2017). Disturbances in norepinephrine neurotransmission in the CNS are increasingly associated with developing psychiatric and neurological diseases (Vazey and Aston-Jones, 2012; Bäuerl et al., 2018), although pathophysiological implication remains limited (Moret and Briley, 2011). The biosynthesis of this neurotransmitter takes place mainly at the adrenal medulla and postganglionic neurons by the multiple enzymatic reactions in which the structural changes of tyrosine, a precursor molecule, to dopamine occurs primarily in the cytoplasm, while the alteration of dopamine to norepinephrine by dopamine β-monooxygenase takes place in the neurotransmitter vesicles (Zahoor et al., 2018). Bacteria such as *Bacillus mycoides*, *Bacillus subtilis*, *Proteus vulgaris*, and *Serratia marcescens* have been reported as norepinephrine-producing microorganisms (Tsavkelova et al., 2000), while *E. coli* K-12, *Bacillus* spp., and *Saccharomyces* spp. have also displayed noradrenalin-producing ability (Shishov et al., 2009; Cryan and Dinan, 2012). Sperandio et al. (2003) reported that norepinephrine is responsible for the quorum-sensing ability of the bacterial population (Sperandio et al., 2003). Wu and Luo (2021) also considered norepinephrine as one of the five main signaling molecules in the classical quorum-sensing system involved in interkingdom communication (Wu and Luo, 2021). The bacterial adrenergic receptors QseC (encoded by the *qseC* gene) and QseE (encoded by *qseE*) are membrane-bound histidine kinases that sense epinephrine and norepinephrine (Kendall and Sperandio, 2016). QseC quorum-sensing sensors have been associated with changes in bacterial motility and activation of virulence genes in several bacteria, including enterohemorrhagic *E. coli* and *Salmonella enterica* serovar Typhimurium (Karavolos et al., 2008; Kendall and Sperandio, 2016). It is documented that bacterial quorum-sensing sensors also sense the host hormones norepinephrine/epinephrine so that they may be interchangeable in the crosstalk between the microbiota and human gut (Li et al., 2019; Wu and Luo, 2021).

Although the related biosynthesis pathway of these neurotransmitters involving the gut microbiome remains unclear, it is assumed that the above bacteria may possess the relevant enzyme, such as dopamine β-monooxygenase needed for converting dopamine into norepinephrine. Shishov et al. (2009) reported that bacterial cells could produce and degrade monoamine neuromodulators *via* enzyme systems that are presumably similar to those found in animals (Shishov et al., 2009).

### 2.3. Neurotransmitter precursors and their biosynthesis pathways

The gut microbiome is primarily known to perform a fundamental function in metabolizing indigestible material consumed by the host, thus contributing to optimum energy production. Accordingly, human colonic bacteria have access to 5–12 grams of proteinaceous material daily. Therefore, amino acids, an essential part of the human diet, serve not only as the basic building blocks of proteins and peptides but also as the precursors to a wide variety of bioactive molecules essential for signaling pathways and metabolic processes. Given the diversity of amino acids and the complex mechanisms involved in metabolic pathways, we will focus here on amino acids that serve as precursors for neurotransmitters.

#### 2.3.1. Tryptophan and its metabolites

As an essential aromatic amino acid, tryptophan is found in several common foods, such as milk, fish, cheese, chocolate, bananas, bread, and wine. It is composed of an indole group and a β carbon. For more than a century, it has been known that certain bacteria can produce amino acids, a trait that has been significantly exploited in the food and feed industry. Since the 1980s, the development of the amino acid industry has been vibrant and has centered primarily on amino acids for feed supplements, which constitute 56% of the total market. The remaining 44% were primarily used in the agriculture, pharmaceutical, food, and cosmetic industries (Lim et al., 2019). A number of studies have indicated that lactic acid bacteria (LAB) possess genes for amino acid synthesis in addition to their well-established proteolytic system. An increasing understanding of the functions and properties of amino acid-producing bacteria has led to increasing commercial interest and diverse commercial applications. LABs are also considered an excellent candidate for amino-acid production for feed supplements (Lim et al., 2019). It is known that some gut bacteria, including *E. coli*, can produce tryptophan, but there is no evidence that bacteria-derived tryptophan contributes significantly to host health (Krautkramer et al., 2021). Since tryptophan is not produced by animal cells, humans must obtain it from an exogenous source through their diet. It has been reported that members of *Clostridium* spp. and *Tannerella* spp. co-occurred with tryptophan biosynthesis and contained genes for tryptophan biosynthetic pathways (Kaur et al., 2019; Valles-Colomer et al., 2019; Aleti et al., 2022). Generally, five enzymes encoded by seven genes (*trpA-F*), typically arranged in a single cluster, are involved in tryptophan biosynthesis in microbes (Crawford, 1989). Gut microorganisms can convert tryptophan to several signaling molecules, including serotonin, melatonin, tryptamine, and other indole derivatives. As mentioned above, tryptophan metabolism is a major pathway leading to the production of serotonin in the gut environment. It is noteworthy that gut-produced serotonin may indirectly impact central serotoninergic pathways, even if they do not cross the BBB, by modulating tryptophan and tryptamine availability (Agus et al., 2018). Some members of the human gut microbiota, such as *Clostridium sporogenes,* have been identified to decarboxylate tryptophan to produce tryptamine, a chemical that modulates host neurological activity (Williams et al., 2014). In addition, tryptophan is also the precursor to melatonin, which acts as an antioxidant and free radical scavenger in microorganisms while having positive effects on human health and could regulate the circadian sleep–wake rhythm if it crosses the BBB. It is noticeable that melatonin is mainly produced in the pineal gland (Danilovich et al., 2021).

In addition, 90% of the circulating tryptophan is metabolized through the kynurenine pathway in the human body (Jenkins et al., 2016). Kynurenine has importance in generating cellular energy in the form of nicotinamide adenine dinucleotide (NAD^+^; Savitz, 2020). In the first step of the kynurenine pathway, tryptophan is converted to N-formylkynurenine by indoleamine 2,3-Dioxgenase 1 and 2 (IDO-1 and IDO-2) and tryptophan-2,3-dioxygenase (TDO), then converted to kynurenine by formamides (Kennedy et al., 2017). Lastly, kynurenine is metabolized into NAD^+^ by different enzymes such as kynurenine aminotransferases (KATs), kynurenine monooxygenase (KMO), and 3-hydroxyanthralinic acid dioxygenase (HAAO; Schwarcz and Pellicciari, 2002). Więdłocha et al. (2021) recently reviewed the current knowledge on the effect of gut microbiota on the kynurenine pathway and their relation with specific psychiatric disorders such as schizophrenia, Alzheimer’s disease, bipolar disorder, depression, autism spectrum disorders, and alcoholism (Więdłocha et al., 2021). Authors mentioned that gut bacteria are capable of synthesizing kynurenine pathway enzymes analogous to TDO, formamidase, KATs, and KMO, which affect this pathway further (Kurnasov et al., 2003; Więdłocha et al., 2021). Synthesis of B6 and B12 vitamins are also dependent on gut microbiome activity. These compounds are cofactors to kynurenine pathway enzymes (Oxenkrug et al., 2013).

Indoles is also one of the derivatives of tryptophan metabolisms. It is documented that the bacterial metabolism of tryptophan generates more than 600 indoles in the gut (Regunathan-Shenk et al., 2022). Indoles are structurally related to neuroactive substances such as serotonin and Lysergic acid diethylamide (LSD). The structural similarity of these compounds has led to increased interest in their potential as neurotoxins. However, studies showed that administration of uremic indoles showed no altering CNS function (Himmelfarb and Sayegh, 2010). Walters and Sperandio (2006) mentioned that indole is considered a bacterial quorum-sensing system in the gut and acts as a signaling molecule. The same authors highlighted that indole could contribute to adapting bacterial cells to nutrient-poor environments where amino acid catabolism is an important energy source (Walters and Sperandio, 2006). A recent study showed that *Lactobacillus reuteri* isolated from murine gut microbiomes metabolize host dietary tryptophan into indole derivatives, kynurenines, and cresol and imidazoles, which may be involved in the regulation of CNS autoimmunity (Montgomery et al., 2022).

#### 2.3.2. Glutamate and its metabolites

Prokaryote and eukaryote organisms produce glutamate as a part of their intra- and inter-kingdom signaling. A portion of the free glutamate in the lumen comes from bacterial synthesis. For instance, several bacteria, such as *Corynebacterium glutamycum*, *L. plantarum*, *L. paracasei*, and *L. lactis,* were reported to produce glutamate (Mazzoli and Pessione, 2016). Although, glutamate plays a fundamental role as an excitatory neurotransmitter in the central nervous system (CNS) and in the enteric nervous system (ENS), where it is synthesized by neurons and glia (Miladinovic et al., 2015). It has been demonstrated that Gram-positive and Gram-negative bacteria use glutamate as a substrate for synthesizing GABA *via* decarboxylation by glutamate decarboxylase (GAD; Tsai and Miller, 2013). Therefore, we mainly considered microbiota-produced glutamate as a precursor for GABA, as mentioned above, and a signaling molecule in this section. A comprehensive evaluation of the microbiome-gut-brain axis and glutamate as a neurotransmitter/neuromodulator has been elegantly reviewed elsewhere (Baj et al., 2019). Authors mentioned that glutamatergic pathways may contribute to interkingdom communication in the gut microbiota (Baj et al., 2019).

Ionotropic (iGlu) and metabotropic (mGlu) glutamate receptors are the two major types of glutamate receptors. Studies have identified at least 100 prokaryotic potassium channels containing putative glutamate binding domains, of which 22 have homology with vertebrate iGlu receptors (Ger et al., 2010). This point allows hypothesizing that glutamate can play a role as inter-bacterial and inter-kingdom signaling molecules and glutamate-producing bacteria can modulate signaling pathways both locally and systemically. There are some evidence that the modulation of glutamatergic receptors along the microbiome-gut-brain axis affects several physiological responses in the brain and the gut, potentially having significant consequences for diseases involving dysfunctions of this communication pathway (Filpa et al., 2016; Mazzoli and Pessione, 2016). It is noteworthy that more investigations are needed to identify gut bacteria able to produce, sense, and respond to glutamate.

Previously, probiotics administered to mice resulted in a long-lasting increase in levels of glutamine/glutamate in the brain, suggesting that the gut microbiome may control enzymatic biosynthesis pathways involved in the production of glutamate in the brain since the BBB impedes the passage of amino acids into the CNS under physiological conditions (Janik et al., 2016). As mentioned above, GABA is synthesized in the gut environment from glutamate through the enzymatic activity of GAD. In addition, the gut microbiome may indirectly affect glutamatergic pathways along the microbiome-gut-brain axis by controlling the metabolic process for L-tryptophan (Agus et al., 2018). It is relevant to mention that decarboxylation of glutamate to GABA is an important survival mechanism for bacteria in the stomach’s extreme acidity (Feehily and Karatzas, 2013).

## 3. Other microbiota-produced neuroactive metabolites

Several metabolites produced by the gut microbiome contribute to the host physiology and homeostasis through, for instance, serving as substrates for reactions or signaling molecules. Although elucidating host-microbiome interactions remains challenging due to the high diversity of produced metabolites and the extent of crosstalk among gut microbes, several actionable microbial targets relevant to host health have been identified through metabolite-focused research (Krautkramer et al., 2021). Here, we mainly discuss only metabolites reported to have mental effects.

### 3.1. Short-chain fatty acids

Extensive research studied the production and metabolism of short-chain fatty acids (SCFAs) by gut microbes. SCFAs are a subclass of fatty acids, ranging from one to six carbon atoms, and they are generated by the gut microbiota fermentation of nondigestible polysaccharides/fibers (Krautkramer et al., 2021). The main route of SCFA production in the colon occurs *via* saccharolytic fermentation of carbohydrates not absorbed in the small intestine, mainly nondigestible polysaccharides/fibers. Butyrate is also formed from amino acid metabolism, and produced SCFAs contribute to the decrease in the pH of the colon (Louis and Flint, 2017). The most common SCFAs found in the human body are acetate, propionate, and butyrate, along with less amount of fumarate, valerate, and caproate, and their levels reach nearly 500–600 mmol per day in the gut depending on the composition and amount of fiber in the diet (Macfarlane and Macfarlane, 2003). In some studies, SCFAs modulated neurotransmitter and neurotrophic factors levels (Silva et al., 2020). Acetate has been shown to alter glutamine, glutamate, and GABA levels and stimulate the production of anorexigenic neuropeptides in the hypothalamus (Frost et al., 2014). Butyrate was also reported with antidepressant properties and effects on social dominance (Hao et al., 2019; Wang, T. et al., 2022). Likewise, propionate, a precursor in lipid biosynthesis, has neuroprotective effects (Hu et al., 2018). In this research, propionate was found to protect against haloperidol-induced neurite lesions and prevent the reduction of neuropeptide Y (Hu et al., 2018). Moreover, SCFAs influence the expression of tryptophan 5-hydroxylase 1 that is responsible for the synthesis of serotonin as well as tyrosine hydroxylase, which takes part in the biosynthesis of dopamine, adrenaline, and noradrenaline; thus, SCFAs play a crucial role in brain neurochemistry by affecting the production of neurotransmitters (Reigstad et al., 2015; Yano et al., 2015; Dalile et al., 2019). Even though the detailed mechanism of their action in the CNS remains unclear, some animal studies have shown that SCFAs have a widespread influence on significant neurological and behavioral processes and may be engaged in important steps of neurodevelopmental and neurodegenerative disorders (Dalile et al., 2019; Fung et al., 2019).

Metagenomic approaches have been widely used to determine individual bacterium responsible for generating SCFAs in the colon. The production routes for propionate, butyrate, and lactate are more conserved and substrate-specific than the acetate production pathways; for instance, limited bacterial genera are involved in propionate production (Reichardt et al., 2014). Many studies have been carried out to identify SCFAs-producing microorganisms and their substrates, and are presented in Table 1. A report listed SCFAs-producing gut microbiomes along with dietary sources used for fermentation (Cheng et al., 2021). The authors found 11 gut commensals that possess a potential capacity to produce SCFAs in the colon, including *Bifidobacterium* spp., *Eubacterium* spp., *Ruminococcus* spp., *Prevotella* spp., *Faecalibacterium* spp., *Collinsella* spp., *Atopobium* spp., *Enterococcus* spp., *Lactobacillus* spp., *Clostridium cluster* XIVa, and *Roseburia* spp. (Cheng et al., 2021). Basson et al. (2016) also provided a list of acetate-, propionate-, butyrate- and lactate-producing gut microbiomes (Basson et al., 2016). It is reported that *Akkermansia muciniphila* is a representative propionate-producing organism (Naito et al., 2018). Moreover, Ze et al. (2012) showed that *Ruminococcus bromii* significantly contributes to butyrate production in the presence of resistant starch in the colon (Ze et al., 2012). Besides, Chang et al. (2021) combined bioinformatics to scan gut-inhabiting *Clostridia* genomes pathways and *in vitro* assay to detect fatty acid amides, revealing that these metabolites might mimic human signaling molecules to modulate their host (Chang et al., 2021). Wang, T. et al. (2022) recently demonstrated that most dominant hosts are characterized by butyrate-producing core microbes, and that colonization of *Clostridium butyricum* alone is adequate to restore the host’s dominance (Wang, T. et al., 2022). In addition, SCFAs commonly have chemical structures similar to the diffusible signal factors (DSF) families. Some Gram-negative bacteria use DSFs as quorum-sensing signals for biofilm formation and virulence. SCFAs, as DSFs mimic, can inhibit bacterial biofilm or other dependent gene expressions in the quorum-sensing system, influencing autoinducer signals (Kumar et al., 2020). Furthermore, SCFAs can be used by other bacteria or pathogens as sources of nutrients or aid colonization, virulence, and invasion. For instance, SCFAs promote adhesion, flagellum growth, and virulence of *Salmonella* Typhimurium by upregulating the expression of T3SS gene (Lawhon et al., 2002).

**Table 1:** SCFAs-producing microorganisms and substrates associated with bacterial fermentation.

SCFAs type	Bacterial strains	Substrate	Potential neuroactivity	Deficiency effect	Ref.
Acetate	*Bacteroides* (*B. thetaiotaomicron*)	Cellulose, hemicellulose, pectin, fructans, mucins, mucopolysaccharides	Cognitive functions	Depletion of acetate-producing bacteria resulted in the reduction of synaptophysin in the hippocampus as well as learning and memory impairments in diabetic mice	Basson et al. (2016), Zheng et al. (2021)
	*Ruminococci*	Celluloses			
	*Bifidobacteria*	Milk oligosaccharides, fructose, lactose			
	*Clostridia*				
	*Proteobacteria* (*Desulfovibrio pigler*)				
	*Eubacteria*				
	*Fusobacteria*				
	*Peptoccocci*				
	*Peptostreptococci*				
	*Propionibacteria*				
	*Veillonella*				
Propionate	*Bacteroides*	Cellulose, hemicellulose, pectin, fructans, mucins, mucopolysaccharides	Effect on anxiety and stress behaviors	Minimal variation in the abundance of butyrate and propionate was observed in the gut of depressed individuals compared to healthy controls; however, antidepressant-like effects of sodium propionate were reported	Liu et al. (2015), Basson et al. (2016), Hoyles et al. (2018), Li et al. (2018)
	*Clostridium* cluster IX				
	*Propionibacteria*				
	*Veilonella*					*Akkermansia municiphilla*	Mucin and mucopolysaccharides
Acetate, propionate, and butyrate	*Faecalibacterium* spp. *Prevotella* spp. *Bifidobacterium* spp. *Eubacterium* spp. *Ruminococcus* spp. *Collinsella* spp. *Atopobium* spp. *Enterococcus* spp. *Lactobacillus* spp. Clostridium cluster XIVa	Pectin, fructans			
	*Roseburia* spp.	Hemi-cellulose, bacterial polysaccharides				Milk oligosaccharides, fructose, lactose
	Butyrate	*Roseburia* spp.				Hemi-cellulose, fructose, fructans	Neuroprotective effects	The long-term supplementation of acetate, propionate, and butyrate in drinking water for chronic cerebral hypoperfusion mice models revealed a positive neuroprotective effect by reducing inflammation and hippocampal neuronal apoptosis following bilateral occlusion of the common carotid artery.	Basson et al. (2016), Xiao et al. (2022)
*F. prusnitztii*									
*E. rectale*									
*E. hallii*									
*R. bromine*									
*Anaerostipes*									
*Ruminococcus bromii*		Ze et al. (2012)						
*Lachnospiraceae*		Plant polysaccharides	Sun et al. (2021)					
Lactate	*Bifidobacterium* spp.	Milk oligosaccharides, fructose, lactose	Antidepressant effect	To the best of our knowledge, no study has examined the relationship between lactate production in the gut microbiome and its deficiency effect. However, there is a well-established interchange of lactate between the periphery and the CNS.	Basson et al. (2016), Caspani et al. (2019)
	*Collinsella aerofaciens*				

### 3.2. Neuroactive peptides

Peptide YY, glucagon-like peptide 1, gastric inhibitory peptide, cholecystokinin, oxytocin, corticotropin-releasing factor, and ghrelin are only found in gut produced by the stimulation of the enteric bacterial microbiome. In the systemic circulation, gut peptides can bind cognate receptors on vagus nerve terminals and immune cells, enabling indirect communication between the gut and the brain. Intestinal microbiome composition influences gut peptide concentrations and enteric signals (Lach et al., 2018). The neuropeptide Y family is the brain’s most abundant family of peptides and is expressed across the gut-brain axis, such as enteric neurons, primary afferent neurons, sympathetic neurons, and several neuronal pathways throughout the brain (Holzer and Farzi, 2014). In the brain, neuropeptide Y, for instance, is expressed by a multitude of neuronal systems in regions spanning from the medullary brainstem to the cerebral cortex. Gut peptides YY and pancreatic polypeptides are mainly released by enteroendocrine cells, where peptide YY is released by the L cells of the ileum and colon in response to food intake. Gut peptides can be activated by their cognate receptors in vagal afferents to signal the brain stem (Latorre et al., 2016). A recent study has identified dipeptides (Phe-Val and Tyr-Val) and their biosynthetic gene clusters in the human microbiome (Cao et al., 2019). These molecules play a critical role in quorum sensing (cell-to-cell communication) to promote the growth of beneficial *Bifidobacterium* and maintain cell density (Hatanaka et al., 2020). A previous study showed that the Phe-Phe produced by *Clostridium* sp. can inhibit host cellular proteins, particularly cathepsins, by chemical modifications causing inflammation (Guo et al., 2017). Another study showed that three quorum sensing peptides (BIP-2, PhrANTH2, PhrCACET1) could selectively penetrate BBB, and two of them influx into the mouse brain (Wynendaele et al., 2015). Since gram-positive bacteria mostly use peptides as signal molecules, this may highlight the potential benefits of probiotics and the human microbiome in depression, anxiety, and stress (Luna and Foster, 2015). This topic is undoubtedly an area of research that requires further exploration.

Other studies showed that some bacterial strains could modulate the expression of gut peptides. For example, Ko et al. (2022) reported that the administration of *L. plantarum* SBT2227 promotes sleep in *Drosophila melanogaster* through the induction of neuropeptide F (a homolog of mammalian neuropeptide Y; Ko et al., 2022). On the other hand, different types of proteases are produced by the gut microbiome, which results in the generation of a large number of peptides during the digestion of food proteins. In the case of simulated gastrointestinal digestion *in vitro*, some studies have shown the production of bioactive peptides (Wu et al., 2021). For instance, Capriotti et al. (2015) showed that hundreds of peptides with various biological activities were produced from soybean proteins in the simulated gastrointestinal digestion. It has been found in other studies that these peptides were stable and remained intact, allowing them to reach their target sites and exert their potential health benefits (Miri et al., 2019; Virgilio, 2019). However, little is known about the interaction mechanism of peptides produced by the gut microbiome and enteroendocrine cells and their interactions with brain physiology.

### 3.3. Bile acids

The liver synthesizes primary bile acids primarily from cholesterol metabolism, a process that is in part mediated and controlled by the gut microbiome. It is thought that microbial enzymes are responsible for deconjugating and dehydroxylation of conjugated primary bile acids to produce secondary bile acids that function as signaling molecules (Wahlström et al., 2016). Due to the possibility that gut bacteria may control the composition of the brain’s bile acid pool, bile acids may serve as a communication link between the gut microbiome and the brain (Monteiro-Cardoso and Corlianò, 2021). It is well-documented that the vagal nerve modulates brain function indirectly through neurotransmitters, which are unlikely to cross the BBB. However, studies demonstrated that bile acids could cross the BBB and are therefore capable of directly signaling through the brain’s bile acids receptors. Still, little is known about the molecular mechanisms involved and the physiological functions of microbiome-derived bile acids in the central nervous system.

### 3.4. Vitamins

**Figure 3:**
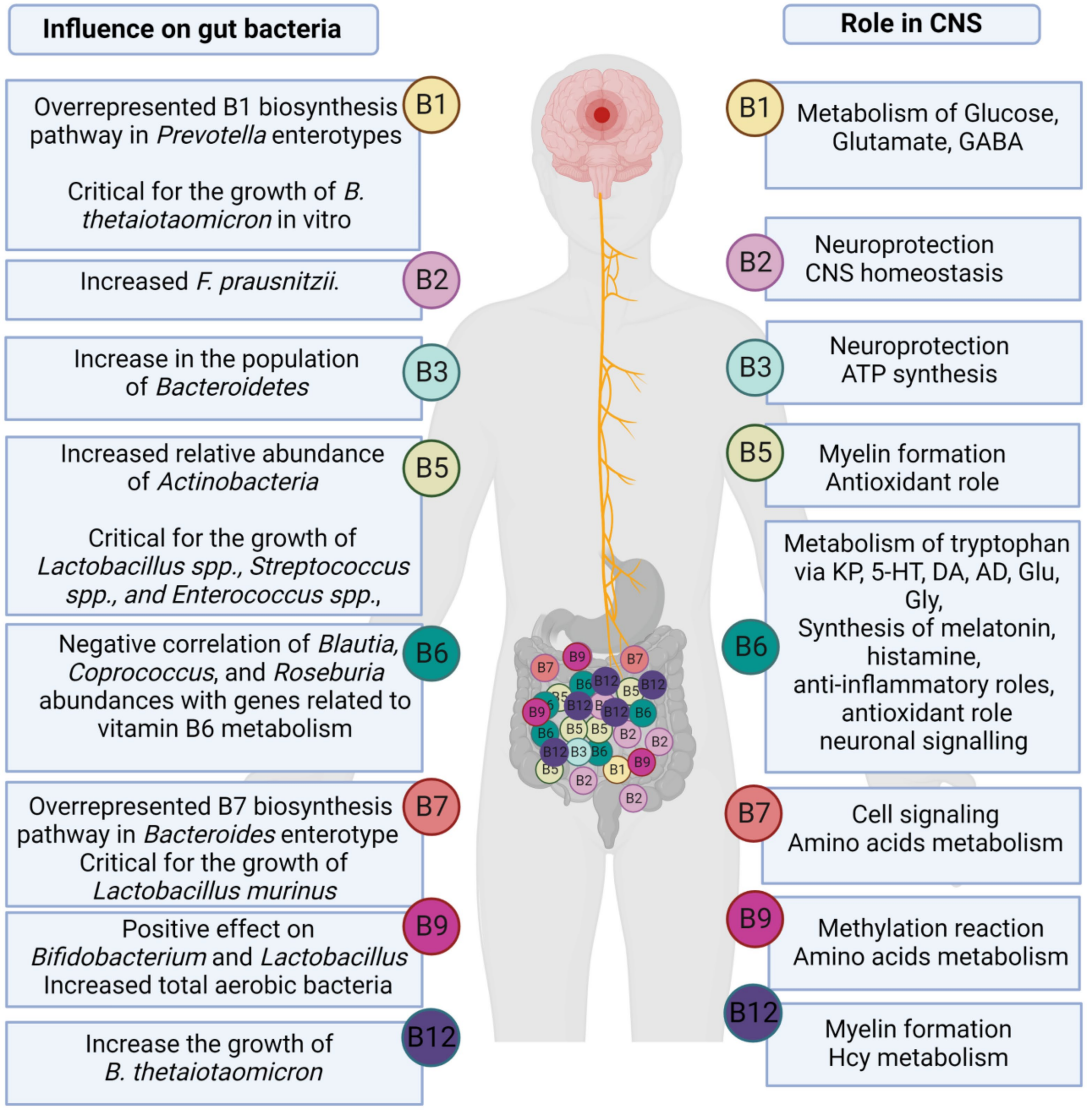
The role of microbially-produced B vitamins in CNS and gut microbiome.

Most gut microorganisms have the ability to synthesize *de novo* and metabolize vitamins, including vitamin K2 (menaquinone), vitamin A (retinol), as well as water-soluble B-vitamins, such as B1 (thiamine), B2 (riboflavin), B3 (niacin), B5 (pantothenic acid), B6 (pyridoxine), B7 (biotin), B9 (Folate), and B12 (cobalamin; Das et al., 2019; Rudzki et al., 2021). Several biochemical processes, such as the metabolism of neurotransmitters, require the B vitamins as coenzymes. Microbial-produced B vitamins and their role in CNS and their effect on gut bacteria are summarized in Figure 3. B vitamins play an important role in neuroprotection, myelin formation, energy production, mitochondrial function, and cellular respiration, as well as exert antioxidant and anti-inflammatory properties (Rudzki et al., 2021). Das et al. (2019) studied the abundance of vitamin biosynthetic gene(s) and consumption of vitamins through uptake transporter(s) using human fecal metagenomic data collected from four different countries (i.e., China, USA, Spain, and Denmark; Das et al., 2019). The authors showed that the range of total gene abundances remained constant across healthy populations in all studied countries. Based on their estimation, 49% of vitamin-related pathways are found in the *Firmicutes* phylum, 19% in the *Proteobacteria* phylum, 14% in the *Bacteroidetes* phylum, and 13% in the *Actinobacteria* phylum (Das et al., 2019; Rudzki et al., 2021). Moreover, a comprehensive analysis of 256 common human gut bacteria genomes revealed that 40–65% of these bacteria could produce some or all of the B vitamins. This prediction was validated by published data in 88% of cases (Magnúsdóttir et al., 2015). It is also important to note that gut microbial metabolism of B vitamins is age dependent. There has been evidence that infant gut microbiomes are enriched for genes involved in *de novo* folate biosynthesis, whereas adult gut microbiomes are enriched for genes involved in folate metabolism and its reduced form tetrahydrofolate (Yatsunenko et al., 2012).

### 3.5. Other potential neurochemical compounds

Recently, Sultan et al. (2022) reported the presence of several neurotransmitter-related compounds or their precursors, such as arachidonyl-dopamine (NADA), gabapentin, and N-acylethanolamines inside gut microbiome-secreted extracellular vesicles (MEVs; Sultan et al., 2022). Dopamine, a representative human neurotransmitter, was also found in these MEVs as a conjugated form with arachidonic acid. N-acylethanolamines (NAEs), such as palmitoyl-ethanolamide (PEA) and linoleoyl-ethanolamide (LEA), have been reported as effective neuroprotective agents (Sun et al., 2007; Schomacher et al., 2008). Also, NADA is an endocannabinoid with widespread physiological and pharmacological activities, including modulation of neuropathic pain, inflammatory hyperalgesia, and immune and vascular systems (Grabiec and Dehghani, 2017). Two potential biosynthetic pathways for NADA have been proposed, though no conclusive evidence exists. First, NADA biosynthesis pathways could involve the conjugation of *N*-arachidonoyl tyrosine to *N*-arachidonoyl-l-DOPA by tyrosine hydroxylase (TH), which would then be converted to NADA by L-amino acid decarboxylase (AADC). Hu et al. (2009) reported the possibility that fatty acid amide hydrolase (FAAH) has the potential to be involved in the direct conjugation of dopamine with arachidonic acid liberated from arachidonoyl-ethanolamide (AEA), the blockade of which significantly decreases *in vivo* the production of NADA (Hu et al., 2009). According to the same authors, FAAH functions either as a rate-limiting enzyme that liberates arachidonic acid from AEA, a conjugation enzyme, or both (Hu et al., 2009). Previous comparative analyses of FAAH enzymes from bacteria, yeast, and mammals showed a strong evolutionary relationship. The alignment of bacterial amidases and mammalian FAAH cDNA confirmed the existence of a highly conserved region known as the signature sequence (Mayaux et al., 1990; Cravatt et al., 1996). This evidence implies the potential presence of genes coding for FAAH enzymes in the gut microbiome, but this has not yet been reported.

## 4. Impact of neuroactive compounds on the gut environment

Neurochemicals, such as GABA, serotonin, dopamine, or their precursors and derivatives, are microbially metabolized by gut commensals and being considered major modulators of the gut environment, including the enteric nervous system (Sarkar et al., 2016). Neuroactive molecules, such as GABA, once secreted into the intestinal environment by bacteria, possibly induce epithelial cells to release molecules that, in turn, modulate neural signaling within the enteric nervous system and consequently signal the brain function and behavior of the host. For instance, *Bifidobacterium dentium* ATCC 27678, a GABA-producing bacterium, was shown to modulate sensory neuron activity in a rat fecal retention model of visceral hypersensitivity, suggesting that GABA-producing bacteria may represent future therapeutics for recurrent abdominal pain and functional bowel disorders (Pokusaeva et al., 2017). The GABA neurochemical was detected in the cytoplasm and brush border of epithelial cells in the rat jejunum and colon (Wang, 2004). The exposure of GABA to epithelial cells selectively stimulated *MUC1* expression in isolated pig jejunum (Braun et al., 2015) and increased the expression of tight junctions and transforming growth factor beta (TGF-β; Sokovic Bajic et al., 2019) while decreasing IL-1β-mediated inflammation *in vitro* (Sokovic Bajic et al., 2019), providing a protective effect against the disruption of the intestinal barrier. GABA-producing bacteria are believed to modulate the gut microbiome and interact with the brain *via* GABAergic signaling *via* vagal afferent neurons (Pokusaeva et al., 2017). The GABAergic system involves GABA receptors, neurons, and enzymes that regulate the immune system to release inflammatory cytokines and attenuate pain. The contribution of the GABAergic system in the pathogenesis of mood disorders is now well-recognized (Northoff and Sibille, 2014; Romeo et al., 2018). Additionally, probiotic bacteria can alter GABA receptor mRNA expression in the brain, which is associated with reduced anxiety and depression (Holzer and Farzi, 2014). Importantly, GABA has also been identified as an essential growth factor that solely can induce the growth of unculturable gut microorganisms (Strandwitz et al., 2019). Indeed, bacteria are known to both produce and consume GABA (Strandwitz et al., 2019). GABA consumption has been studied less than GABA production, however, Feehily and Karatzas (2013) found that GABA is converted to succinate for use in the TCA cycle (Feehily and Karatzas, 2013). Dover & Halpern also described GABA as a source of nitrogen and carbon in *E. coli* (Dover and Halpern, 1972). GABA-producing bacteria also could modulate the gut microbiome structure and metabolism. In our recent study, we have shown the potential of *Bifidobacterium animalis*, *Lactobacillus delbrueckii* subsp. *bulgaricus*, and *Streptococcus thermophilus*, three GABA-producing bacteria, to promote gut health (Mousavi et al., 2022). While these GABA-producing probiotic candidates had no change in gut microbiome diversity, *ex-vivo* supplementation induced an increase of the *Bacteroidetes*, a key gut population having anti-inflammatory properties (Mousavi et al., 2022). The relative abundance of *Bacteroides*, a major GABA-producing genus in the gut, was also negatively correlated with depression-associated brain signatures (Strandwitz et al., 2019), indicating a significant role of microbiota-derived GABA in brain functionality. Also, *Bacteroides* spp. were linked with higher levels of serotonin, and myoinositol, which is pivotal in maintaining signaling between the enteric and central nervous systems (Mudd et al., 2017). Likewise, Mason et al. (2020) have reported depletion of *Bacteroides* in depression and anxiety (Mason et al., 2020). The oral administration of *B. fragilis* reduced gut permeability, microbiome dysbiosis, and several behavioral abnormalities in a mice model of autism spectrum disorder (ASD), thus highlighting the potential of microbial interventions for the treatment of gut microbiota-mediated neurological disorders (Hsiao et al., 2013).

Interestingly, Wang, J. et al. (2022) demonstrated that pollutants-treated zebrafish could be rescued from the disorder of intestinal peristalsis by using an exogenous treatment containing 100 μg/L of serotonin (5-hydroxytryptophan). They also suggested that *Lactobacillus rhamnosus* GG could normalize gut motility *via* increasing serotonin secretion (Wang, J. et al., 2022). It is estimated that 90–95% of the body’s serotonin is located within the gastrointestinal tract. The gut microbiome produces a significant amount of serotonin (Kelly et al., 2015). At the same time, these levels of serotonin affect the gut microbiome. Researchers found that increased levels of serotonin promote the colonization of gut bacteria. In other studies, dopamine and norepinephrine have also been shown to affect the gut microbiome. For instance, *E. coli* grows more rapidly when dopamine and norepinephrine are present. It also exhibits an increase in biofilm formation, motility, and virulence in the presence of norepinephrine (Yano et al., 2015; Strandwitz, 2016). In addition to the ability to produce histamine, gut bacteria could degrade it. It is important to note that if more histamine is produced than is degraded, this could create symptoms of histamine intolerance. Eventually, this results in gut inflammation (Shulpekova et al., 2021). Moreover, microorganisms that produce SCFAs in the gut have been demonstrated to suppress gut motility. These findings support the theory that the microbiome participate in gut motility regulation through gut-to-brain signaling (Kelly et al., 2015; Muller et al., 2020). The understanding of the effect of neuroactive compounds on gut microbiome composition and activity is still limited despite significant efforts. Some recent studies mentioned that serotonin has a quorum-sensing effect on probiotic *Enterococcus faecium* NCIMB10415 and *Campylobacter jejuni*, a pathway that can modulate their behavior and subsequent interaction with the gut epithelium (Lyte et al., 2021; Scardaci et al., 2022). Due to the critical role of gut microorganisms in the production of neuroactive compounds and mental health, further research in this area is necessary.

## 5. Transport mechanisms of gut microbiota-produced neuroactive metabolites to the brain

It has long been assumed that gut-produced neurotransmitters, such as GABA, are unlikely to cross the BBB, but the investigations that have built this paradigm are often conflicting and vary widely in their used methods (Boonstra et al., 2015). However, recent research points out that gut microbiota-derived neurometabolites may cross intestinal barriers and reach distal organs, such as the brain. A fecal transplant from lean to obese individuals illustrated such gut microbiome-host interplay, which resulted in increased plasma levels of GABA (Kootte et al., 2017). For instance, gut microbiota-derived GABA is potentially transported through different pathways to the brain. The intestinal GABA absorption may occur *via* the transcellular pathway with the support of the relevant carrier proteins, and Nacher et al. (1994) reported that GABA could share a transporter with β-alanine in rat intestine models. GABA in the plasma can enter the BBB through GABA transporters such as GABA transporter types 1, 2, 3, and 4 (GAT1, GAT2, GAT3, and GAT4, respectively), which are also widely distributed to other organs, including the liver and kidneys (Nacher et al., 1994). The plasma membrane GABA transporters in the brain play a crucial role in maintaining the extracellular GABA level around the synapse (Liu et al., 2015). The GABA transporter is an active voltage-dependent system in which the inward electrochemical gradient of Na^+^ ions significantly affects the activity of the GABA transporter instead of ATP (Scimemi, 2014). Furthermore, the GABA transporter shows a weak micromolecular affinity to GABA molecules and requires Cl^−^ ions in the extracellular matrix (Scimemi, 2014). Still, the exact transportation mechanism of GABA from the intestinal tract to the brain is not well understood. Likewise, most neurotransmitters, such as dopamine, norepinephrine, and acetylcholine, present in blood circulation cannot penetrate the BBB due to the absence of relevant transporters (Chen et al., 2021). However, the precursors of the above neurotransmitters, such as tyrosine and tryptophan, can penetrate BBB; thus, they can be transferred to the corresponding cells and used to synthesize corresponding neurotransmitters in the brain.

The SCFAs produced by the gut microbiota-mediated fermentation of fiber are absorbed through the colonocytes *via* monocarboxylate transporters (MCTs) and sodium-coupled MCTs (SMCTs), which are known as active transport (Vijay and Morris, 2014). SCFAs are transported *via* MCT1 transporters in an H^+^-dependent (electroneutral manner), while they are also transported through the electrogenic and sodium-dependent SMCTs, known as SCFA anion transport (Stumpff, 2018). Most SCFAs introduced into the colonocytes are metabolized by entering the citric acid cycle in the mitochondria to produce ATP and energy (Schönfeld and Wojtczak, 2016). However, some portions of SCFAs in the colonocytes are not metabolized, which leads to their introduction into the portal circulation, used as an energy source for hepatocytes, except for acetate, which is not metabolized in the liver (Schönfeld and Wojtczak, 2016). This indicates that only a limited amount of colon-derived SCFAs is allowed to enter the systemic circulation and other organs and tissues; namely, only 36, 9, and 2% of gut-derived acetate, propionate, and butyrate, respectively, reach the blood plasma and peripheral tissues (Boets et al., 2015). Bloemen et al. (2009) reported that the respective average levels of acetate, propionate, and butyrate in the portal blood of humans were 260, 30, and 30 μM (Bloemen et al., 2009). However, the penetration capacity of SCFAs in the BBB has not been well investigated to date, indicating that more research is needed to better understand the effects of gut microbiota-derived neuroactive metabolites on brain functions.

**Figure 4:**
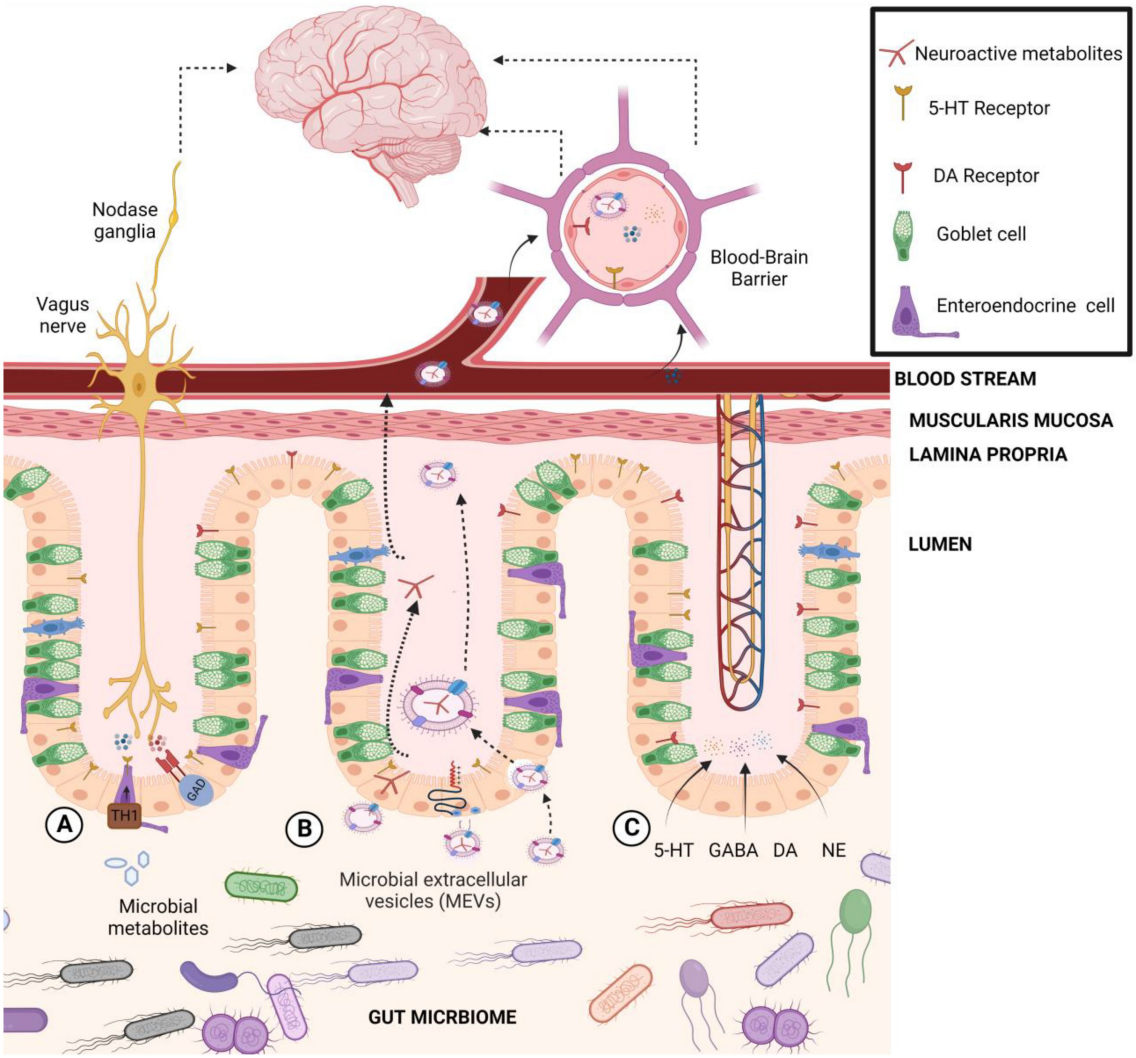
The transportation pathways of gut microbiota-derived neuroactive compounds to the brain. **(A)** Indirect transportation: gut microbiome regulates or induces host biosynthesis of neurotransmitters in cells like serotonin (5-HT) through tryptophan hydroxylase 1 (Tph1) or GABA through glutamate decarboxylase (GAD). **(B)** Microbial extracellular vesicle transportation: MEVs may bind to the cell receptor and deliver their contents to the host cell, activate a cell response, or be fully incorporated into the host cell’s cytoplasm. **(C)** Direct transport: Microbially modulated neurotransmitters could interact with receptors or circulate systemically to reach the blood–brain barrier.

Recently, secreted microbiota extracellular vesicles (MEVs) have been proposed as a potential new carrier for the transportation of gut microbiota-derived neuroactive compounds to the brain (Sultan et al., 2021, 2022; Figure 4). Accumulating evidence suggests that MEVs are significant mediators in the intercellular signaling mechanism that could be an integral part of microbiome-host communications (Sultan et al., 2021). MEVs are small membrane-bound phospholipid vesicles that encase a spectrum of biologically active molecules (i.e., proteins, mRNA, miRNA, DNA, carbohydrates, and lipids) that protect them from lytic enzymes and RNases in the extracellular environment (Al-Nedawi et al., 2015) and facilitate their horizontal transfer across both short and distant locations, such as the brain (Choi et al., 2015; Sultan et al., 2021). For instance, *Akkermansia muciniphila*-produced extracellular vesicles were reported to induce serotonin secretion in both the colon and hippocampus of mice, suggesting MEVs’ potential as signaling molecules in the gut–brain axis (Yaghoubfar et al., 2020). Besides, MEVs may cross intestinal barriers and reach distal organs, such as the liver and adipose tissues, inducing insulin resistance and glucose intolerance (Choi et al., 2015). A reported increased level of systemic LPS-positive bacterial MEVs in humans with intestinal barrier dysfunction provides evidence of their capacity to reach the systemic circulation (Tulkens et al., 2020) and deliver and elicit various immunological and metabolic responses in different organs, including the brain. From another point of view, the phospholipid nature of MEVs itself may directly influence neuronal function under stress-related conditions (Donoso et al., 2020). For instance, *Lactiplantibacillus plantarum*-secreted extra vesicles exhibited an antidepressant-like effect in chronic restraint stress-treated mice (Choi et al., 2019). MEVs released by *Bacteroides fragilis* contain GABA and its intermediates α-ketoglutarate and glutamate as part of their content (Zakharzhevskaya et al., 2017). MEVs containing neuroactive compounds from *B. fragilis* may explain the observation of a previous study that showed the oral administration of this bacteria reduced gut permeability, microbiome dysbiosis, and several behavioral abnormalities in a mice model of autism spectrum disorder (ASD; Hsiao et al., 2013). Also, *Bacteroides*, a significant GABA-producing genus in the gut, was linked with higher levels of serotonin, and myoinositol, which is pivotal in maintaining signaling between the enteric and central nervous systems (Mudd et al., 2017). The relative abundance of *Bacteroides* was negatively correlated with depression-associated brain signatures (Strandwitz et al., 2019), indicating a significant role of microbiome-secreted GABA in brain functionality. Likewise, Mason et al. (2020) have reported depletion of *Bacteroides* in depression and anxiety (Mason et al., 2020). Recently, metabolomics profiling of MEVs content isolated from human gut microbiome revealed presence of a wide array of embedded metabolites, including neurotransmitter-related compounds such as arachidonyl-dopamine (NADA), gabapentin, glutamate and N-acylethanolamines (Sultan et al., 2022). The same authors reported that gut *Bacteroides* isolates (*B. finegoldii*, *B. faecis,* and *B. caccae*) produce high GABA levels (4.5–7 mM range) in supernatants, and importantly, GABA was detected inside secreted microvesicles at 2.2–4 μM. Such vesicles can transfer their cargo to the host cells such as Caco-2, RIN14B, and hCMEC/D3 cells, which showed capacity to internalize labeled MEVs through an endocytic mechanism (Sultan et al., 2022). These results provided novel insights on the shuttle role of MEVs for neuroactive molecules to the brain as a new signaling mechanism in microbiota-gut-brain axis communications. MEVs should be considered of utmost importance as delivery vehicles for host neuroactive compounds to the intestinal mucosa and other organs in the body such as the brain, thus, affecting the host’s mental health.

## 6. Conclusion and future perspectives

One of the most intriguing and controversial topics in microbiome research is the relationship between gut microbial metabolism and mental health. Accumulating evidence showed that the gut microbiome produces a broad spectrum of neuroactive compounds, including neurotransmitters and their precursors, highlighting a potential involvement in neuroendocrinology-based mechanisms. One of the key challenges facing this field is the identification of neuroactive compounds originating from the host rather than the gut microbiome, which can be challenging due to complex biological communications between the gut microbiome and the brain. It is also difficult to determine the extent to which gut microbial metabolism directly influences central nervous system activity. This limitation may be attributed partly to the lack of a clear understanding of the general rate at which microbial molecules are transported into the brain. Indeed, the direct effects of microbial metabolites on the central nervous system function are difficult to distinguish from other communication pathways (such as immunological or neuronal pathways) that could confound *in vivo* studies. Some of these neuroactive compounds can travel through portal circulation to interact with the host’s enteric nervous system, influence metabolism, or affect local neuronal cells of the ENS and afferent pathways of the vagus nerve that signal directly to the brain. When neurotransmitters cannot pass the BBB, their bacterial precursors do (such as tyrosine and tryptophan); thus, they can be located in the corresponding cells and synthesized into neurotransmitters in the brain. However, recent studies highlighted that secreted microbiome extracellular vesicles are potential new carriers for the transportation of gut microbiota-derived neuroactive compounds to the brain. In addition, most of the studies focusing on these relationships have relied heavily on simplified animal models, which cannot adequately simulate the complexity of the mechanism of microbial-produced neuroactive. Therefore, more studies on the mechanism, biosynthesis, absorption, and transportation of gut microbiota-derived neurotransmitters to the brain are needed. More analytical and statistical frameworks are needed to acquire and integrate multi-omics data types for a systematic approach to this extensively complex system. As described above, gut microbial neuroactive metabolites have various health-promoting effects. Despite recent research progress, multiple questions surrounding this field of gut neuromicrobiology remain unsolved. Indeed, there is a limited understanding of how gut microbes orchestrate the microbiome-gut-brain axis, a prerequisite for developing evidence-based microbiota-targeted interventions. Future research needs to progress from phenomenological studies to a mechanistic understanding of the microbiome-host dialogue and how these microbes impact host neurobiological functions. Future studies integrating metabolomic and metagenomic profiles with functional and behavioral outcomes will help us bridge this gulf of understanding toward translation into specific microbiota-targeted interventions. While further investigations remain necessary before the possibilities for evidence-based therapeutic applications, this review provided an overview of the biosynthesis and transport of gut microbiome-derived neurotransmitters and their precursors and interplays with the microbiome-gut-brain axis.

## Author contributions

SM, JY, and RH designed and wrote the first draft of this article. SM, SA, and RH reviewed and edited the manuscript. All authors contributed to the article and approved the submitted version.

## Funding

This study was supported by a grant from the Natural Sciences and Engineering Research Council of Canada (NSERC; No. RGPIN-2018-06059) and a Weston Family Foundation grant through its Weston Family Microbiome Initiative. JY and SM were supported by the Nutrition and Mental Health postdoctoral fellowship, University of Ottawa.

## Conflict of interest

The authors declare that the research was conducted in the absence of any commercial or financial relationships that could be construed as a potential conflict of interest.

## Publisher’s note

All claims expressed in this article are solely those of the authors and do not necessarily represent those of their affiliated organizations, or those of the publisher, the editors and the reviewers. Any product that may be evaluated in this article, or claim that may be made by its manufacturer, is not guaranteed or endorsed by the publisher.
